# Diagnosing and managing a giant primary cutaneous malignant melanoma of the lower limb

**DOI:** 10.1093/jscr/rjac409

**Published:** 2022-09-19

**Authors:** Ryan Faderani, Stephen R Ali, Amit Nijran, Thomas D Dobbs, Richard Karoo

**Affiliations:** Faculty of Medicine, University College London, UK; Welsh Centre for Burns and Plastic Surgery Morriston Hospital, Swansea, UK; Welsh Centre for Burns and Plastic Surgery Morriston Hospital, Swansea, UK; Reconstructive Surgery and Regenerative Medicine Research Group, Institute of Life Sciences, Swansea University Medical School, Swansea, UK; Welsh Centre for Burns and Plastic Surgery Morriston Hospital, Swansea, UK; Welsh Centre for Burns and Plastic Surgery Morriston Hospital, Swansea, UK; Reconstructive Surgery and Regenerative Medicine Research Group, Institute of Life Sciences, Swansea University Medical School, Swansea, UK; Welsh Centre for Burns and Plastic Surgery Morriston Hospital, Swansea, UK

## Abstract

We present a woman who was referred to our plastic surgery unit with a suspected squamous cell carcinoma following a 3-year history of an enlarging mass on her thigh. Surprisingly, histopathological assessment confirmed the diagnosis of nodular malignant melanoma measuring 77×77×54 mm with a Breslow thickness of 52 mm, making it the largest recorded lower limb primary cutaneous malignant melanoma in the UK.

## INTRODUCTION

Malignant melanoma is the fifth most common cancer in the UK, with an incidence rising faster than many other cancers [1]. Stage I disease is treatable under local anaesthetic with a 5-year survival of 99.6% [2]. Delays in presentation can be deadly, with stage II and III disease having 5-year survival figures of 80.4 and 70.6%, respectively [2]. This article highlights the challenges of diagnosing and managing such a case in the context of the COVID-19 pandemic.

## CASE REPORT

An 84-year-old woman was referred to our plastic surgery unit for assessment and management of a large lesion on her right upper thigh that had been slowly growing over 3 years. The patient denied any history of previous skin cancer and had a minimal history of sun exposure. She had no other specific risk factors for skin malignancy. Over the last 6 months, the lesion developed a foul odour, began bleeding and started to enlarge, at which point the patient reluctantly sought medical attention from her General Practitioner. She decided to keep the lesion hidden from her family during this symptomatic period as she cited worry about undergoing any surgery in the context of COVID-19. At this point, she was referred to the regional plastic surgery service as an urgent suspected cancer 2-week-wait pathway [3]. Her medical history consisted of lymphoedema secondary to chronic venous insufficiency, atrial fibrillation and raised body mass index (44.8 kg/m^2^). Her World Health Organisation performance status was 3.

**Figure 1:**
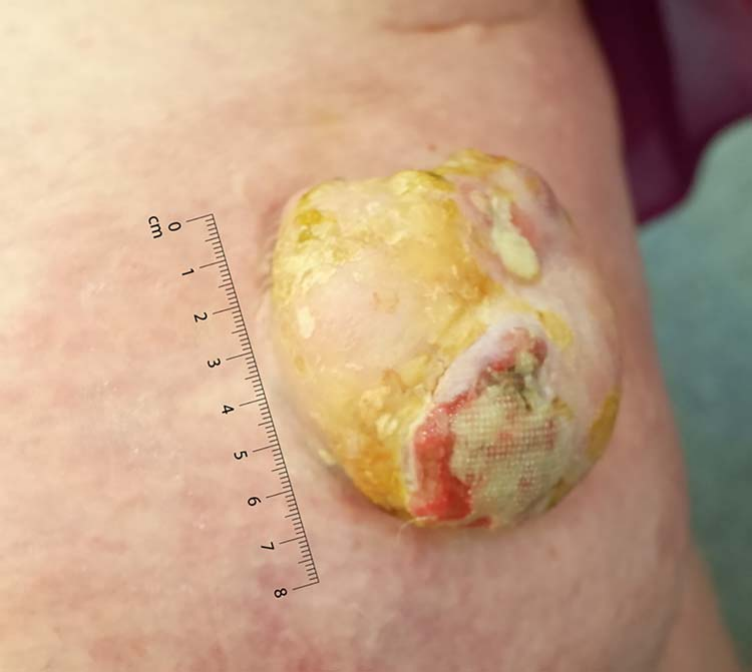
Large left lower limb ulcerating lesion at the time of presentation, to scale.

The lesion was examined using a dermatoscope by the operating surgeon. On examination, she was Fitzpatrick skin type II. There was a large, exophytic, fungating and ulcerating lesion on the right medial thigh (Fig. 1). The lesion was not fixed to any deep structures clinically. There was no palpable lymphadenopathy in the inguinal lymph node basin nor was there any evidence of visible or palpable in-transit disease. A head-to-toe skin check did not reveal any other suspicious skin lesions. Following assessment, the primary differential was that of a squamous cell carcinoma. Two weeks later, under local anaesthetic, the lesion was excised with a 1 cm peripheral margin. The defect was resurfaced with a split thickness skin graft.

**Figure 2:**
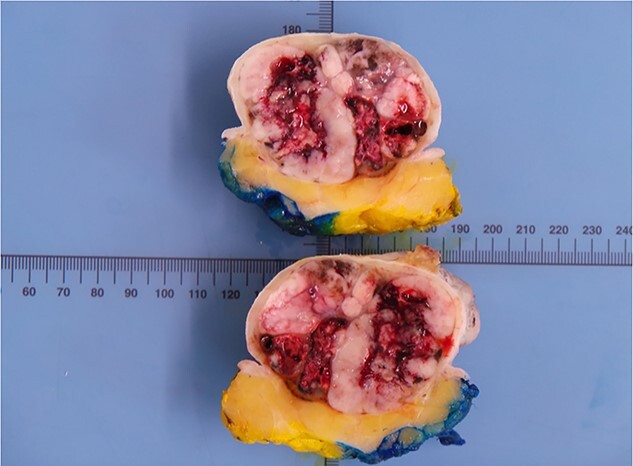
Macroscopic image of the lesion following bisection.

**Figure 3:**
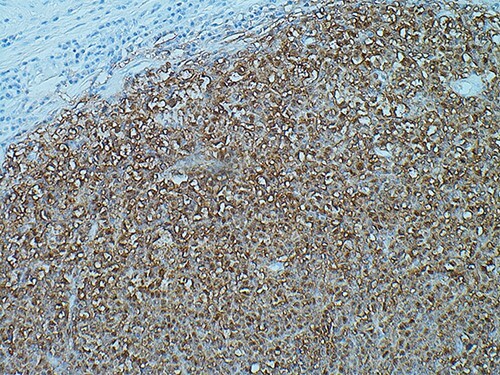
SOX10 immunohistochemistry showing nuclear stain staining confirming the diagnosis of malignant melanoma, magnification ×200.

Histological analysis revealed that this lesion was an invasive, ulcerated nodular malignant melanoma in the vertical growth phase with a Breslow thickness of 52 mm, Clark level of 5 and mitotic count of 5–10/mm^2^ measuring 77×77×54mm macroscopically, pathologically staged at pT4BpNxpM0. Bisection of the lesion revealed areas of haemorrhage and necrosis (Fig. 2). Tissue blocks revealed large areas of ulceration of the surface epidermis with no residual or visible melanocytic precursor in the intact epidermis on the vicinity of the tumour. The lesion was completely excised with a peripheral margin of 10 mm and a deep margin of 34 mm. Lymphovascular invasion was present but perineural invasion, tumour infiltrating lymphocytes and regression were all absent. No microsatellites or in transit metastases could be identified. Immunohistochemical analysis revealed the lesion was positive for S100, Melan-A, SOX10 and BCL-2. (Fig. 3) The tumour was BRAF positive. Based on these findings, the tumour was staged at IIC.

Following discussion at the SSMDT, a 1 cm wide local excision (totalling 2 cm, including the excision biopsy clearance) and sentinel lymph node biopsy (SLNB) was recommended in addition to computed tomography (CT) staging of the thorax, abdomen, pelvis and magnetic resonance imaging (MRI) of the brain [3]. Both CT and MRI did not identify any evidence of metastatic disease despite the T4 stage of the tumour. SLNB was declined by the patient. The case was discussed at further SSMDT again where histopathological review of the slides noted large ulceration of the surface epidermis with no residual or visible melanocytic precursor in the intact epidermis on the vicinity of the tumour. In these pathological findings and the absence of any previous cutaneous melanomas or non-cutaneous melanomas in other sites, it was concluded that this lesion represented a giant primary cutaneous malignant melanoma. Clinical surveillance was instigated at 3-monthly intervals. Following 1-year follow-up, the patient remains well without any signs of recurrence.

## DISCUSSION

There is no formal definition of giant melanomas; however, the mean size of the maximum dimension has been reported as 13 cm (5–25 cm) and Breslow thickness as 45 mm (4–100 mm), whereas the other authors confine the term to melanomas of over 10 cm in diameter, irrespective of depth [4]. Breslow thickness is an important prognostic tool which is incorporated into the TNM staging for melanoma [5]. ‘Thick melanomas’ have a Breslow thickness of >4 mm and a 5-year survival of 50% [6]. In the literature, the largest metastatic lesion of melanoma measures 35 × 29 × 25 cm [7]. Meo *et al*. reported only 16 documented cases of giant cutaneous melanomas as of 2014 [4]. The head and neck and upper limb are the two most common anatomical areas where one is more likely to have a giant cutaneous melanoma [4].

One of our key challenges in this case was in ascertaining whether this lesion represents a primary cutaneous melanoma or metastatic disease. This dilemma often arises when a connection to the overlying epidermis cannot be ascertained on histopathology, and thus it is unknown whether the tumour originates from the skin or from elsewhere. In many giant cutaneous melanomas, extensive epidermal erosion makes it difficult to determine an epidermal connection and thus a primary cutaneous derivation.

However, there are several clinical and histopathological features that can be used to help distinguish between primary lesions and cutaneous metastases of malignant melanoma. First, primary lesions will typically display features of epidermotropism as well as junctional activity, whereas metastatic lesions rarely present with this due to limited intraepidermal extension [8–10]. Second, along with epidermotropism, one would expect to observe an inflammatory reaction with the presence of inflammatory infiltrate in primary lesions, which again is rarer for metastases [8, 9]. Furthermore, although rare, a total regression of a primary tumour is possible; however, we would expect to see an abnormal skin lesion with characteristic areas of fibrosis secondary to chronic inflammation [8, 9]. Following SSMDT peer review and reference to these clinical and histopathological hallmarks (epidermotropism evident on S100 staining, no history of previous cutaneous or non-cutaneous mucosal melanoma or current metastasis and no evidence of a regressed skin lesion), it was determined that the histology of the lesion in the current case represented a primary ulcerated nodular melanoma. Finally, on reviewing the literature, a large proportion of giant melanomas reported have occurred as primary lesions without a precursor.

Surgical resection with suitable margins is the only curative treatment for malignant melanoma [3, 6]. There is no consensus on the specific management of giant malignant melanomas due to the limited case numbers and heterogenicity of cases [11]. To the best of our knowledge, there are only two other reported cases of giant melanomas located on the lower limb and this is the first case reported in the United Kingdom (UK) (Table 1) [7, 12].

**Table 1:** Summary of giant limb melanomas described in the current literature

**Author**	**Location**	**Age**	**Gender**	**Size (mm)**	**Breslow thickness (mm)**	**LN status**	**M status**	**Centre**
Benmeir *et al*. [7]	Lower limb	37	F	350 × 290 × 250	–	Y	N	Israel
Kiyak *et al*. [10]	Lower limb	56	F	180 × 160 × 50	48	Y	Y	Turkey
Our case	Lower limb	84	F	77 × 77 × 54	52	N	N	UK
del Boz *et al*. [13]	Upper limb	29	F	200 × 150 × 70	70	-	Y	Spain
Tseng *et al*. [14]	Upper limb	63	M	230 × 210 × 60	75	Y	Y	USA
Tseng *et al*. [14]	Upper limb	88	M	100 × 80 × 30	31	N	Y	USA
Honeyman and Wilson [15]	Upper limb	57	F	140 × 70 × 120	70	N	N	UK

LN, lymph node status; M, metastases.

This case highlights the exigencies of diagnosing and managing a giant melanoma in the context of the COVID-19 pandemic. Clinicians should be cognizant to late presentations of both non-melanomatous skin cancer and melanoma cases in light of the psychosocial impact the pandemic may have on delays in the cancer diagnostic journey.

### Learning Points

1. To consider a giant cutaneous primary melanoma as a differential diagnosis in patients with large fungating cutaneous lesions.
2. With a significant increase in delayed presentations as a result of the pandemic, it is important for clinicians to consider the psychosocial impact of the pandemic on patients.
3. Although melanomas with larger Breslow thickness are typically associated with poor prognosis, this may not be the case in some giant cutaneous melanomas.

## REPORTING STANDARDS

CARE guidelines.

## References

[1] Cancer Research UK . Melanoma skin cancer statistics. 2020. https://www.cancerresearchuk.org/health-professional/cancer-statistics/statistics-by-cancer-type/melanoma-skin-cancer#heading-Zero (12 September 2020, date last accessed).

[2] Office for National Statistics . Cancer survival by stage at diagnosis for England. 2020. https://www.ons.gov.uk/peoplepopulationandcommunity/healthandsocialcare/conditionsanddiseases/datasets/cancersurvivalratescancersurvivalinenglandadultsdiagnosed (12 September 2020, date last accessed).

[3] National Institute for Heath and Care Excellence (NICE) . Melanoma: Assessment and Management. UK: NICE guideline, 2015. 26334080

[4] di Meo N , Stinco G, Gatti A, Errichetti E, Bonin S, Albano A, et al. Giant melanoma of the abdomen: case report and revision of the published cases. Dermatol Online J 2014;20:13030/qt4pp2825w. 25046463

[5] Amin MB . AJCC Cancer Staging System, 8th edn. USA: American Joint Commitee on Cancer, 2017.

[6] Marsden JR , Newton-Bishop JA, Burrows L, Cook M, Corrie PG, Cox NH, et al. Revised UK guidelines for the management of cutaneous melanoma 2010. J Plast Reconstr Aesthet Surg 2010;63:1401–19. 20728418 10.1016/j.bjps.2010.07.006

[7] Benmeir P , Neuman A, Weinberg A, Sucher E, Weshler Z, Lusthaus S, et al. Giant melanoma of the inner thigh: a homeopathic life-threatening negligence. Ann Plast Surg 1991;27:583–5. 1793246 10.1097/00000637-199112000-00013

[8] David Elder, Rosalie Elenitsas . Benign pigmented lesions and malignant melanoma. In: Elder D (ed). Lever’s Histopathology of the Skin, 8th edn. Wolters Kluwer Health, USA 1997, 719–31.

[9] DE Vries E , Bray F, JW C, Cerroni L, Ruiter DJ, et al. Melanocytic tumors. In: Skin Tumours, 10th edn. WHO Classification of Tumours, 68–9

[10] Gerami P , Shea C, Stone MS. Angiotropism in epidermotropic metastatic melanoma: another clue to the diagnosis. Am J Dermatopathol 2006;28:429–33. 17012920 10.1097/01.dad.0000204761.40199.3f

[11] Honeyman CS , Wilson P. Patient with giant upper limb melanoma presenting to a UK plastic surgery unit: differentials and experience of management. BMJ Case Rep Geneva, Switzerland: WHO; 2016;2016: bcr2015212253. 10.1136/bcr-2015-212253 PMC4746506 26838295

[12] Kiyak MV , Yesilada AK, Sevin KZ, Usta U. Giant malignant melanoma: a case report. Acta Chir Plast 2012;54:59–61. 23565846

[13] del Boz J , García JM, Martínez S, Gómez M. Giant melanoma and depression. Am J Clin Dermatol 2009;10:419–20. 19824743 10.2165/11311050-000000000-00000

[14] Tseng WW , Doyle JA, Maguiness S, Horvai AE, Kashani-Sabet M, Leong SP. Giant cutaneous melanomas: evidence for primary tumour induced dormancy in metastatic sites? BMJ Case Rep 2009;2009:bcr0720092073. 10.1136/bcr.07.2009.2073 PMC3027359 21977058

[15] Honeyman CS , Wilson P. Patient with giant upper limb melanoma presenting to a UK plastic surgery unit: differentials and experience of management. BMJ Case Rep 2016;2016:bcr2015212253. 10.1136/bcr-2015-212253 PMC4746506 26838295

